# Cytokines in Clinical and Experimental Transplantation

**DOI:** 10.1155/S0962935194000566

**Published:** 1994

**Authors:** Ann C. T. M. Vossen, Huub F. J. Savelkoul

**Affiliations:** Department of Immunology Erasmus University PO Box 1738 Rotterdam 3000 DR The Netherlands

## Abstract

Allograft rejection is a complex process, which requires interactions between different cell types and a variety of soluble factors, such as cytokines. In this review we discuss the role of cytokines in the induction and effector phases of the rejection process and in the induction and maintenance of allospecific graft tolerance. Furthermore, we discuss the feasibility of clinical graft function monitoring by measuring cytokines and the possibilities for intervention in the cytokine network in order to inhibit graft rejection and eventually obtain graft acceptance.


Mediators of Inflammation 3, 403–410 (1994)

ALLOGRAFT rejection is a complex process, which requires interactions between different cell types and a variety of soluble factors, such as cytokines. In this review we discuss the role of cytokines in the induction and effector phases of the rejection process and in the induction and maintenance of allospecific graft tolerance. Furthermore, we discuss the feasibility of clinical graft function monitoring by measuring cytokines and the possibilities for intervention in the cytokine network in order to inhibit graft rejection and eventually obtain graft acceptance.

**Key words:** Cytokines, Graft rejection, Monoclonal antibody treatment, Th1–Th2 subsets, Tolerance

### Cytokines in clinical and experimental transplantation

**Ann C. T. M. Vossen and Huub F. J. Savelkoul[CA]**

Department of Immunology, Erasmus University, PO Box 1738, 3000 DR Rotterdam, The Netherlands

[CA] Corresponding Author

### Introduction

Cytokines are proteins, which act as soluble mediators and regulators of immune responses. They perform their actions by binding to specific membrane-bound receptors and act in a paracrine or autocrine fashion. Different cell types, not only leukocytes, can produce the same cytokine or react to the same cytokine. So, one cytokine can have a range of differential effects on various different target cells (pleiotropy). Furthermore, two different cytokines can have the same effect (redundancy). Cytokines are able to positively or negatively influence the production and function of one another. The complexity of the cytokine network should be kept in mind, while interpreting experimental or clinical findings as discussed in this review.

One of the immune responses in which cytokines are considered to play an important role is allograft rejection. The process of rejection has been studied carefully and starts to become more clear.[1–3] During the induction phase of rejection, T-cell receptor (TcR) recognition of the foreign MHC molecule results in T-cell activation, expression of the interleukin (IL)-2 receptor (R) and production IL-2, which in an autocrine fashion induces clonal expansion of the activated T cells. During T cell activation, soluble (s)IL-2R are released.[4] It is thought that $CD4^+$ T lymphocytes are essential for initiation of graft rejection and $CD8^+$ T cells are more important in the effector phase of rejection.[5] In this phase, production of IL-2 and other cytokines induces influx and proliferation of $CD8^+$ cytotoxic T cells, natural killer (NK) cells and macrophages. This inflammatory process will lead to tissue destruction and dysfunction of the graft. Cytokines can influence this process at several levels (Table 1). IL-1, produced by macrophages, acts as an accessory signal from the antigen presenting cell (APC) for T cell proliferation.[6,7] IL-2 is a growth factor for T lymphocytes, induces T lymphocyte cytotoxicity and stimulates NK cell activity.[8] Interferon (IFN)-γ activates macrophages, cytotoxic T cells and NK cells and induces increased expression of MHC class I and II molecules in this way increasing the allogenicity of the graft.[9] Tumour necrosis factor (TNF)-α increases the MHC class I expression and activates neutrophils.[10–12] Apart from these activities, cytokines may have direct cytotoxic effects on the grafted tissue.[13]

Since $CD4^+$ T cells are of such importance for graft rejection, T-helper (Th)1–Th2 subsets have been studied for their potential role in regulating the rejection process. Both in mice and in human, different Th cell clones have been described, which can be distinguished by the different sets of cytokines they produce.[14,15] Th1 cells produce IL-2, IFN-γ and

**Table 1.** Cell sources of cytokines relevant in transplantation

| | CTL[a] | Th1 | Th2 | NK | B cell | Monocytes/ macrophages | Other[b] |
|---|---|---|---|---|---|---|---|
| IL-1 | | | | | + | + | + |
| IL-2 | + | + | | | | | |
| IL-4 | | | + | | | | |
| IL-5 | | | + | | | | |
| IL-6 | | | + | | | + | + |
| IL-10 | | | + | | | + | |
| IFN-γ | + | + | | + | | | |
| TNF-α | + | + | + | + | | + | + |
| TNF-β | + | + | | + | | | + |

[a]CTL, cytotoxic T lymphocytes.
[b]Other cells producing these cytokines are fibroblasts, epithelial cells etc.

© 1994 Rapid Communications of Oxford Ltd lymphotoxin (LT, also known as TNF-β), while Th2 cells produce IL-4, IL-5, IL-6 and IL-10. Other cytokines, such as IL-3 and TNF-α, are produced by both Th subsets.

Different functions have been assigned to the different Th subsets. Th1 cells mediate delayed-type hypersensitivity (DTH), anti-viral and anti-tumoral cytotoxic effects, while Th2 cells provide help for B cell differentiation and antibody formation. These two Th subsets are able to influence each others cytokine production and proliferation. IFN-γ, produced by Th1 cells, inhibits Th2 cell proliferation and cytokine production.[16] IFNγ also antagonizes the activity of IL-4 in B cell activation and isotype production.[14] The Th2 cytokine IL-10, inhibits the production of Th1 cytokines while IL-4 inhibits Th1 cell proliferation.[17,18] Since cellular cytotoxicity plays an important role in allograft rejection, Th1 cells are thought to dominate in this process. On the other hand, the suppression of these allospecific Th1 cells by Th2 cells could be a mechanism of graft acceptance. Altered cytokine gene expression and production during graft rejection provides a logical target to redirect the observed shifts in the Th1–Th2 balance interfering with the cytokine network.

To further elucidate the role of cytokines in clinical and experimental allograft rejection and survival, we first focus on the detection of cytokines during the rejection process and during graft acceptance. Then we describe the approaches that have been used to interfere in the cytokine network and future prospects for therapeutic intervention.

### Detection of cytokines during graft rejection

There are two reasons for detecting cytokines during allograft rejection. The first is to find a reliable and preferably non-invasive method to monitor graft function. If certain cytokines are found to be early predictors of acute rejection this enables us to start rejection therapy as soon as possible and to monitor the success of this therapy. The second reason is to learn more about which cytokines play a role in the rejection process and therefore might be good targets for immunomodulation. We think that therapeutic intervention in the earliest (induction) phase of rejection is preferable to intervention in the effector phase in order to redirect the immune response and achieve long-term allograft survival. Therefore, we consider cytokines appearing in the induction phase of more importance to serve as targets for immunomodulation than cytokines appearing late in rejection.

The advantage of animal models for graft rejection is that proper controls, including syngeneic grafts and non-grafted tissue can be examined for cytokine expression. Furthermore, in general, no immunosuppressive drugs are being used in these models.

In the clinical setting, immunosuppressive drugs are routinely used for the prevention of organ transplant rejection. Immunosuppressive drugs like cyclosporin A (CsA) and glucocorticoids can inhibit TNF and IL-6 production,[19–21] while the anti-CD3 mAb OKT3 and anti-thymocyte globulin (ATG) can increase serum IL-6 and TNF-α levels.[22–24]

Table 2 summarizes the cytokines described to be involved in allograft rejection.

*Detection of systemic cytokines:* In the clinical setting a lot of effort has been made to find a single or few cytokines in the serum, urine or bile, that permit monitoring graft rejection. In kidney, liver or lung transplantation, serum levels of IL-6,[23,25,26] TNF-α,[27–29] IL-2, and sIL-2R[30–34] have been shown to increase in relation to graft rejection. Serial monitoring was necessary, since not the absolute levels of IL-6 or sIL-2R, but rather a rise of these levels indicated the development of rejection.[25,26,31,35] However, the measured cytokine levels have low sensitivity and specificity regarding rejection diagnosis. TNF-α, IL-2 and sIL-2R levels were found to be elevated in some cases of stable graft function.[29–31,36] IL-6, TNF-α and sIL-2R levels were increased in the early postoperative period and elevations of IL-6, TNF-α or sIL-2R levels were found during episodes of infection.[23,26,27,30–34] Yoshimura *et al.*[23] suggested that serum IL-6 levels or even better the ratio of IL-6 to CsA trough value may discriminate rejection from CsA nephrotoxicity. Possibly, measurements of TNF-α[29] or IL-2[30] in the urine of renal allograft recipients or IL-6[37] in the bile, as shown in a rat hepatic allograft model, will have more value in discriminating allograft rejection from infection or non-immunological graft dysfunction. These studies demonstrate that systemic cytokine measurements are not reliable enough to differentiate rejection from infection. Therefore, they cannot be used for monitoring and histological tissue analysis remains the standard for diagnosis of rejection. Whether these cytokines are involved in the mechanism of allograft rejection, as has been suggested in a few of the above-mentioned studies, is in our opinion doubtful. Based on their paracrine and autocrine activity, the detection of cytokines in the periphery is not likely to reflect the local activities within the graft. It is much more likely that cytokines

**Table 2.** Detection of cytokines implicated in allograft rejection

| | Cytokines | References |
|---|---|---|
| Systemic | IL-2, sIL-2R, IL-6, TNF-α | 23, 25–35 |
| *In situ* animal models | IL-2, IL-4, (IFN-γ), (TNF-β) | 44, 45 |
| *In situ* clinical transplantation | IL-2, IL-5, IL-6, IFN-γ, TNF-α | 46–50 |

that are systemically present, such as TNF-α and IL-6 are involved in the inflammatory process of an ongoing rejection process and are rather nonspecific markers in the serum. Interference with these cytokines could diminish the development of inflammation, but a more preferable target for immunomodulation would be those particular cytokines, that are involved in the induction phase of the local rejection process in order to create a new immunological balance and graft acceptance.

In situ *detection of cytokines during graft rejection:* In order to get more insight into which cytokines are involved in the rejection process, cytokines have been detected in the graft in experimental and clinical settings. For *in situ* detection of cytokines several techniques have been used. Northern blot analysis, reverse transcription (RT)-PCR and *in situ* hybridization were used to detect cytokine mRNA within the graft. In sponge matrix allografts, i.e. sponges containing allogeneic cells, the exudate was tested for cytokine proteins. Furthermore, the local production of cytokines was investigated using immunohistochemistry or cultures of graft-infiltrating cells (GIC). All of these techniques have their own advantages and disadvantages. For example, if cytokine mRNA is to be detected, one cannot conclude that the protein is also present, since the production of cytokines can be regulated at post-transcriptional level. *In situ* hybridization indicates the localization of cytokine production and is also rather sensitive, but is a difficult and time-consuming technique. RT-PCR is an extremely sensitive method, but this method gives no information about the cell-type that produces the detected cytokines. Immunohistochemistry is able to demonstrate the cytokine-producing cells. However, most cytokines are not retained in the cytosol, but are directly secreted after translation. This may cause false negative results. On the other hand, it is difficult to discriminate between the cytokine producing cells and the target cells for these cytokines. Especially interpretations concerning Th1–Th2 imbalances should be made very carefully. This also counts if cytokines are detected in supernatants of cultured graft infiltrating cells. It has been shown that culture conditions, such as added cytokines, can dictate the outcome of the cytokine pattern produced.[38–42] The analysis of mRNA of cultured cells is also unreliable, since manipulating the cells can cause not only induction of a particular cytokine mRNA, but also the level of instable cytokine mRNA to decline.[43]

Two studies in a murine cardiac allograft model[44,45] used the extremely sensitive reverse transcription PCR method to detect cytokine mRNA in normal tissue, isografts and allografts at several days after grafting. Their results were quite similar. The cytokines that were detectable could be divided into three groups. The first group of cytokines can be detected in normal tissue, isografts and allografts and consists of IL-1β, IL-5, IL-6, TNF-α and TGF-β. The second group, including IL-1α and IL-3, is expressed in both isografts and allografts. These two groups seem to represent cytokines which are induced in response to the grafting procedure and are not correlated with the rejection process itself. The last and probably the most important group consists of IL-2 and IL-4. These cytokines are only present in allografts, which suggests they play a role in allograft rejection. However, the two studies show inconsistent data on two cytokines, TNF-β and IFN-γ. Dallman *et al.*[44] showed that TNF-β can only be detected in allografts, while Morgan *et al.*[45] also detected this cytokine in isografts. IFN-γ, however, was only found in allografts by Morgan *et al.*, while Dallman *et al.* detected IFN-γ mRNA in normal tissue and in isografts as well, though the levels were lower than in the allografts. This difference in cytokine pattern remains unexplained. Interestingly, Dallman *et al.* found that IL-2, IL-4 and TNF-β, the cytokines that are expressed only in the allografts, were all transcribed transiently, their expression being maximal at 4–5 days after transplantation. Thereafter the expression declined before clinical signs of rejection appeared, suggesting these cytokines play a role in the initiation of rejection.

In the clinical setting *in situ* detection of cytokines in rejecting allografts has been compared with grafts without signs of rejection. *In situ* hybridization of human renal biopsies showed that normal kidney tissue or biopsies from patients with stable graft function do not significantly express IL-6, TNF-α or IFN-γ transcripts.[46] During acute rejection, however, the grafts showed significant levels of IL-6 mRNA, but not TNF-α or IFN-γ mRNA. The IL-6 mRNA was expressed in many different cell types, such as glomerular cells, tubular epithelium and vascular endothelium. The significance of the IL-6 production by these nonimmune cells remains unclear. Using the same technique on irreversibly rejected kidney grafts, TNF-α mRNA could be detected in macrophage-like infiltrating cells.[47] Immunohistochemistry showed that these cells actually produced TNF-α protein. Glomerular cells and tubular epithelium cells probably were target cells for TNF-α, since TNF-α protein could be detected, but no TNF-α mRNA was found in these cells. The results of mRNA detection during allograft rejection may be influenced by the organ investigated. In human liver allografts IL-1β, IL-4, IL-6 and TNF-α mRNA was expressed in rejecting allografts and in grafts without signs of rejection.[48] Only IL-5 mRNA was associated with liver graft rejection. All the above-mentioned data are derived from grafts, in which the rejection process had developed quite far, as graft dysfunction was already apparent, and the rejection sometimes even irreversible. So, the cytokines detected may play a role in the graft rejection, but may also be rather nonspecific mediators of tissue inflammation and destruction. Two studies, using RT-PCR on fine-needle aspirates, were able to detect IL-2 mRNA[49] and IFN-γ mRNA[50] in samples, taken before clinical rejection. Since the expression was transient, sequential analysis is required to use this method to predict graft rejection.

*Detection of cytokines during allograft acceptance:* In animal models, the induction of allograft tolerance has succeeded by using several different protocols, such as a preoperative donor-specific transfusion (DST) or anti-CD4 monoclonal antibody (mAb) treatment.[51–53] Determination of the changes in the cytokine profile in these accepted grafts compared with rejecting allografts, may clarify the mechanism by which this state of tolerance is maintained and may indicate ways to manipulate the immune system in order to obtain tolerance. Strangely, intragraft events, which have been ascribed to cytokines, such as mononuclear cell infiltration, allospecific cytotoxic T cells and elevated expression of MHC class I and II antigens, are still apparent in tolerized grafts[54] and renal allografts with stable graft function.[55]

In a rat kidney allograft model, in which tolerance was induced by DST, graft infiltrating cells from tolerant rats were unable to produce IL-2 *in vitro*, expressed lower levels of the IL-2R and showed lower proliferation in response to IL-2 than cells from untreated rats.[56] In a comparable model, tolerized rat heart allografts showed a lower expression of IL-2 and IFN-γ mRNA and a different kinetics of these messages than rejecting allografts.[57] Strong evidence that the downregulation of IL-2 or IFN-γ mRNA plays a role in the development of graft tolerance is provided by the fact that simultaneous administration of IL-2 or IFN-γ and the DST abrogates the tolerizing effect. It is still unclear whether this downregulation of Th1 cytokines is the mechanism of tolerance induction or whether Th2 cells play a role in suppressing these Th1 cytokines.

After induction of tolerance, using several different strategies, it has been shown that IL-2 and IFN-γ mRNA were downregulated, whereas IL-4 and IL-10 (Th2 cytokines) mRNA expression remained at the same level or even increased.[58] However, there is no proof that the source of these cytokines is indeed T lymphocytes. Recently, it has been shown, that IL-10 mRNA is detectable in many organs of a normal mouse and is expressed at the same level in nude mice or SCID mice, suggesting independence of T and B cells.[59]

Anti-CD4 mAb treatment has resulted in graft acceptance in several animal models. Partial depletion of CD4+ T cells in mice resulted in a higher expression of IL-4 mRNA and a lower expression of IFN-γ mRNA, as detected by *in situ* hybridization in purified CD4+ T cells.[60] In other studies, using anti-CD4 mAb, long term renal allograft survival and abrogation of accelerated cardiac rejection were accompanied by diminished IL-2 expression and preserved IL-4 expression, suggesting that the Th1–Th2 dichotomy does play a role in immunosuppression.[61,62] Additional evidence is provided by the finding that adoptive transfer of a bm12-specific Th2 cell line to B6 mice resulted in prolongation of bm12 skin grafts, whereas BALB/c third party grafts were rejected.[63] It is interesting to note that IL-4 can be detected both early in the rejection process[44] and during graft acceptance. This finding awaits further clarification.

### Strategies interfering with cytokine effects

CsA and FK506 are important immunosuppressive drugs, that are widely used for preventing organ allograft rejection. It is known for several years now that their immunosuppressive mechanism is based on inhibition of T cell signal transduction pathways leading to activation of cytokine gene expression.[64] Besides these established regimens, alternative ways to interfere with cytokine effects have been studied (Table 3).

*Monoclonal or polyclonal antibodies:* Since cytokines have been shown to be associated with allograft rejection, several strategies have been studied to interfere with cytokine function in order to prolong graft survival.

Monoclonal and polyclonal antibodies against cytokines have been shown to prolong allograft survival in several animal models. Anti-IFN-γ mAb blocked MHC class II disparate skin graft rejection in mice, but had no effect on MHC class I disparate skin graft rejection.[65] Anti-IL-2,[66] anti-TNF-α and anti-LT antibodies[67–69] were able to prolong rat cardiac allograft survival, when given as the only therapy. In the same model, anti-IFN-γ antibodies alone failed to prolong graft survival compared with untreated controls.[70,71] However, in combination with CsA, anti-IFN-γ antibodies prolonged allograft survival in a synergistic way.[70,72] This synergistic action with CsA, that permits lowering of the CsA dose, thereby decreasing its potential nephrotoxicity, is also seen using anti-TNF-α antibodies.[68,69] Furthermore, it has been shown that combining anti-LT with anti-TNF-α

**Table 3.** Strategies interfering with cytokine effects

| Modulator | Targets | References |
|---|---|---|
| CsA, FK506 | IL-2 | 64 |
| Antibodies | IL-2, IFN-γ, TNF-α, TNF-β, IL-2R+ cells | 65–69, 76–85 |
| Cytokine conjugated toxins | IL-2R+ cells | 86, 87 |
| Soluble receptors | IL-1, IL-4 | 88, 89 |
| Cytokines (IL-4, IL-10) | Th2 subset | 98, Dallman *et al.* |

mAb ameliorates graft survival, compared with anti-TNF-α mAb treatment alone.[68]

Although these antibody treatments directed against single cytokines have some effect, graft survival times are not impressive. One of the reasons for this finding could be the property of cytokines to mediate local, i.e. in the graft, short-distance effects. Therefore the local antibody concentration might not be sufficient to neutralize its target cytokine. However, there is evidence that the neutralizing antibodies indeed reach the graft, since $^{125}$I-labelled anti-IL-2 mAb was detectable in the graft.[66] Furthermore, local effects of the mAb, such as diminished mononuclear cell infiltration, no expression of MHC class I antigens and modification of immunohistologic staining pattern of TNF-α, were readily visible.[67,69,72] Another explanation for the disappointing results with cytokine-directed antibodies is the redundancy of the cytokine system. Neutralizing the activity of one cytokine probably makes another take over. The striking redundancy of the cytokine system was again demonstrated by the finding, that IL-2 and IL-4 knock-out mice were less affected in the development and function of their T cell system than expected.[73,74]

Another approach to interfere in the cytokine network, by using mAb directed against the IL-2R (CD25), has been shown to be more successful. Not only the number of publications on the use of this mAb, but also the fact that this treatment is already used in clinical trials, are indicative for its success. Anti-IL-2R mAb therapy differs from anti-cytokine mAb therapy in that it acts on IL-2R bearing cells and not just on soluble proteins. Since high-affinity IL-2R is only expressed on activated T cells and not on resting T cells, this approach seems more specific than other established immunosuppressive therapies using ATG, OKT3 or CsA.[75] The mechanism of the anti-IL-2R induced immunosuppression is still not clear. Depletion of IL-2R$^+$ cells, modulation of the IL-2R or blocking of the IL-2-IL-2R interaction have been proposed to play a role.[76–79] In animal models anti-IL-2R mAb have been used to inhibit GVHD and organ allograft rejection.[76,79–81] Prospective clinical trials have shown that anti-IL-2R treatment is equally effective as ATG for the prevention of renal allograft rejection.[82–84]

The use of mouse or rat mAb in clinical transplantation is hampered by the development of human anti-antibodies, that subsequently lead to high clearance rate of the mAb and abrogation of their effect. Therefore, 'humanized' mAb have been produced, combining the rodent complementarity-determining regions with constant regions and framework of human antibodies. A 'humanized' anti-IL-2R mAb, that was less immunogenic and had a longer half-life than its murine form, significantly prolonged cardiac allograft survival in cynomolgus monkeys.[85] As 'humanized' mAb were being developed, molecular engineering offered an alternative approach for selectively attacking IL-2R$^+$ cells. Cytotoxic substances, such as *Pseudomonas* exotoxin (PE) or *Diphtheria* toxin were coupled to IL-2, thereby targeting and killing IL-2R$^+$ cells. IL-2-PE40 and DAB486-IL-2 were able to inhibit allograft survival in animal models.[86,87]

*Soluble cytokine receptors:* In biological fluids of both animals and humans, cytokine binding proteins have been found, that later appeared to be soluble forms of cytokine receptors, like sIL-2R, sIL-4R and sTNFR. They generally have the same binding affinity for their ligand as the membrane receptors and therefore are able to competitively inhibit cytokine binding to membrane receptors and subsequently their effects on target cells. Soluble cytokine receptors are considered to be naturally occurring cytokine inhibitors and have the advantage of higher affinity and being non-immunogenic over neutralizing mAb. Their potential as therapeutic agents in inflammatory disease and sepsis has been shown. In a cardiac allograft model in mice, sIL-1R and sIL-4R have been shown to somewhat prolong the allograft survival.[88,89]

The efficacy of treatment with soluble cytokine receptors is based on scavenging the relevant cytokine. Therefore, it is crucial to have the soluble cytokine receptor present in the serum with a long half-life. The linking of two sTNFR molecules to the Fc portion of a single human IgG1 molecule resulted in a dimeric form of sTNFR with significantly higher affinity for TNF than the monomeric sTNFR. Moreover, this complex is detectable in serum during 4 – 5 days, being significantly increased over unbound sTNFR. This dimeric sTNFR was very effective in *in vivo* neutralizing endogenous TNF and protecting mice from lethal endotoxaemia.[90,91] Since anti-TNF mAb have been shown to be effective in prolonging allograft survival,[67,69] this agent might have a similar or even more potent effect on allograft rejection. Using soluble cytokine receptors, one should keep in mind that they are also capable of acting as cytokine carriers, protecting them from proteolytic cleavage, prolonging their half-live in the circulation and therefore having an agonistic instead of an antagonistic effect.[92] The ratio in the presence of the cytokine and its soluble receptor probably determines the biological outcome.

*Strategies for Th1–Th2 'skewing':* The assumption that Th1 cells are responsible for rejection, whereas Th2 cells may act as suppressor cells and induce graft acceptance has led to the idea that 'skewing' of the Th1–Th2 ratio towards Th2 dominance might inhibit graft rejection. *In vitro* studies have shown that cytokines can direct the differentiation of Th cells to one of the subsets. In the presence of IFN-γ Th1 cell development is enhanced,[38] whereas IL-4 enhances the development of Th2 cells.[39,40,93] IL-10 does not seem to direct bulk cultures towards Th2 cytokine producing cells, though anti-IL-10 mAb did induce Th1-like cells.[93] Treatment of allograft recipients with IL-4 or IL-10 could have the same 'skewing' effect. Furthermore, IL-10 not only inhibits the production of Th1 cytokines, but also has anti-inflammatory properties by inhibiting IL-1, IL-6 and TNF-α synthesis by macrophages.[94] *In vivo* interference in the dominance of one of the Th subsets has already succeeded in a number of inflammatory models. Giving anti-IL-4 mAb before or within the first week of *Leishmania major* infection rendered a susceptible mouse strain resistant to the parasite,[95] but anti-IL-10 mAb had no effect. The other way around, IL-4 promoted the Th2 response to the parasite, but did not render the infected recipient susceptible.[96] Anti-IFN-γ mAb did render *Trypanosoma cruzi* resistant mice susceptible to the infection.[97] In an allogeneic graft model, the use of anti-IFN-γ or anti-IL-2 mAb has not been very effective in prolonging allograft survival, as we described earlier. There have been two groups reporting on the effect of administration of Th2 cytokines, IL-4 and IL-10, on allogeneic responses. Though cardiac allograft rejection (M. Dallman, personal communication) and enlargement of the draining lymphnode[98] were inhibited, no tolerance was induced. Furthermore, systemic administration of these cytokines could have some drawbacks, such as stimulation of B lymphocytes, antibody-production and increased incidence of infections. Also, there is evidence that systemic administration of cytokines, in this case IFN-γ, may lead to a downregulation of endogenously produced cytokine.[99]

*Prospects for therapeutic strategies:* Strategies directed against a single cytokine in the form of mAb, soluble cytokine receptors or other agents are not likely to be successful in inhibiting graft rejection. Molecular engineered proteins, on the other hand, such as the dimeric form of sTNFR, may be useful to inhibit the intragraft inflammation and systemic effects of graft rejection. Other than in parasitic models, systemic administration of cytokines will probably not be able to interfere with the local processes leading to graft rejection. The increasing knowledge on requirements for T cell activation and the different signalling pathways leading to cytokine production and T cell subset activation, should enable us to interfere with this process in order to establish allospecific tolerance. T cell activation requires besides TcR-MHC interaction costimulatory signals coming from interaction of cell surface molecules on the APC and the T cells.[100,101] Indeed, *in vitro* studies have shown that TcR signalling in the absence of costimulatory signals results in T cell anergy.[102,103] Furthermore, this anergy can be induced in Th1 clones but not in Th2 clones.[104–106] The CD28-B7 interaction seems to play a critical role in the costimulation of T cells.[101,107] *In vivo* blocking of this interaction inhibits cardiac allograft rejection[108] and induces long term survival of pancreatic xenografts.[109] Further studies in *in vitro* and animal models for tolerance should lead to strategies resulting in anergy of allospecific Th1 cells and induction of allospecific 'suppressor' Th2 cells and thereby to allospecific tolerance.

### Concluding remarks

Cytokines are involved in the allograft rejection process. However, the relevance of systemic cytokine measurements in order to predict graft rejection is limited, since elevated cytokine levels are found both during rejection and infection. In the induction phase of rejection, a major role is probably played by IL-2 and IFN-γ together with or in balance with IL-4. During the effector phase, many different cytokines may mediate the inflammation. The local rejection process is not easily inhibited by systemic administration of anti-cytokines or cytokines. Intervention in intercellular signalling may lead to a new immunological balance and a state of graft tolerance, based on Th1 anergy and/or Th2 suppression.

### References

1. Hayry P. Mechanisms of rejection. *Curr Opin Immunol* 1989; **1**: 1230–1235.
2. Colvin RB. Cellular and molecular mechanisms of allograft rejection. *Annu Rev Med* 1990; **41**: 361–375.
3. Wecker H, Auchincloss HJ. Cellular mechanisms of rejection. *Curr Opin Immunol* 1992; **4**: 561–566.
4. Rubin LA, Nelson DL. The soluble interleukin-2 receptor: biology, function, and clinical application. *Ann Intern Med* 1990; **113**: 619–627.
5. Strom TB. The cellular and molecular basis of allograft rejection: what do we know? *Transplant Proc* 1988; **20**: 143–146.
6. Kaye J, Gillis S, Mizel SB, *et al.* Growth of a cloned helper T cell line induced by a monoclonal antibody specific for the antigen receptor: interleukin 1 is required for the expression of receptors for interleukin 2. *J Immunol* 1984; **133**: 1339–1345.
7. Durum SK, Schmidt JA, Oppenheim JJ. Interleukin 1: an immunological perspective. *Annu Rev Immunol* 1985; **3**: 263–287.
8. O'Garra A. Interleukins and the immune system 1. *Lancet* 1989; **i**: 943–947.
9. O'Garra A. Interleukins and the immune system 2. *Lancet* 1989; **i**: 1003–1005.
10. Shalaby MR, Aggarwal BB, Rinderknecht E, Svedersky LP, Finkle BS, Palladino MAJ. Activation of human polymorphonuclear neutrophil functions by interferon-gamma and tumor necrosis factor. *J Immunol* 1985; **135**: 2069–2073.
11. Collins T, Lapierre LA, Fiers W, Strominger JL, Pober JS. Recombinant human tumor necrosis factor increases mRNA levels and surface expression of HLA-A,B antigens in vascular endothelial cells and dermal fibroblasts *in vitro*. *Proc Natl Acad Sci USA* 1986; **83**: 446–450.
12. Tracey KJ, Vlassara H, Cerami A. Cachectin/tumour necrosis factor. *Lancet* 1989; **i**: 1122–1126.
13. Maessen JG, Buurman WA, Kootstra G. Direct cytotoxic effect of cytokines in kidney parenchyma: a possible mechanism of allograft destruction. *Transplant Proc* 1989; **21**: 309–310.
14. Mosmann TR, Coffman RL. TH1 and TH2 cells: different patterns of lymphokine secretion lead to different functional properties. *Annu Rev Immunol* 1989; **7**: 145–173.
15. Romagnani S. Human TH1 and TH2 subsets: doubt no more. *Immunol Today* 1991; **12**: 256–257.
16. Gajewski TF, Fitch FW. Anti-proliferative effect of IFN-gamma in immune regulation. I. IFN-gamma inhibits the proliferation of Th2 but not Th1 murine helper T lymphocyte clones. *J Immunol* 1988; **140**: 4245–4252.
17. Fiorentino DF, Bond MW, Mosmann TR. Two types of mouse T helper cell. IV. Th2 clones secrete a factor that inhibits cytokine production by Th1 clones. *J Exp Med* 1989; **170**: 2081–2095.
18. Mosmann TR, Moore KW. The role of IL-10 in crosssregulation of TH1 and TH2 responses. *Immunol Today* 1991; **12**: 49–53.

19. Nguyen DT, Eskandai MK, DeForge LE, *et al.* Cyclosporin A modulation of tumor necrosis factor gene expression and effects *in vitro* and *in vivo*. *J Immunol* 1990; **144**: 3822–3828.
20. Beutler B, Çerami A. Cachectin: more than a tumor necrosis factor. *N Engl J Med* 1987; **316**: 379–385.
21. Yoshimura N, Kahan BD, Oka T. The *in vivo* effect of cyclosporine on interleukin-6 gene expression in renal transplant recipients. *Transplant Proc* 1991; **23**: 958–960.
22. Abramowicz D, Schandene L, Goldman M, *et al.* Release of tumor necrosis factor, interleukin-2, and gamma-interferon in serum after injection of OKT3 monoclonal antibody in kidney transplant recipients. *Transplantation* 1989; **47**: 606–608.
23. Yoshimura N, Oka T, Kahan BD. Sequential determinations of serum interleukin 6 levels as an immunodiagnostic tool to differentiate rejection from nephrotoxicity in renal allograft recipients. *Transplantation* 1991; **51**: 172–176.
24. Debets JM, Leunissen KM, van Hooff HJ, van der Linden CJ, Buurman WA. Evidence of involvement of tumor necrosis factor in adverse reactions during treatment of kidney allograft rejection with antithymocyte globulin. *Transplantation* 1989; **47**: 487–492.
25. Van Oers MH, Van der Heyden AA, Aarden LA. Interleukin 6 (IL-6) in serum and urine of renal transplant recipients. *Clin Exp Immunol* 1988; **71**: 314–319.
26. Kita Y, Iwaki Y, Demetris AJ, Starzl TE. Evaluation of sequential serum interleukin-6 levels in liver allograft recipients. *Transplantation* 1994; **57**: 1037–1041.
27. Maury CP, Teppo AM. Raised serum levels of cachectin/tumor necrosis factor alpha in renal allograft rejection. *J Exp Med* 1987; **166**: 1132–1137.
28. Imagawa DK, Millis JM, Olthoff KM, *et al.* The role of tumor necrosis factor in allograft rejection. I. Evidence that elevated levels of tumor necrosis factor-alpha predict rejection following orthotopic liver transplantation. *Transplantation* 1990; **50**: 219–225.
29. McLaughlin PJ, Aikawa A, Davies HM, *et al.* Evaluation of sequential plasma and urinary tumor necrosis factor alpha levels in renal allograft recipients. *Transplantation* 1991; **51**: 1225–1229.
30. Simpson MA, Madras PN, Cornaby AJ, *et al.* Sequential determinations of urinary cytology and plasma and urinary lymphokines in the management of renal allograft recipients. *Transplantation* 1989; **47**: 218–223.
31. Colvin RB, Preffer FI, Fuller TC, *et al.* A critical analysis of serum and urine interleukin-2 receptor assays in renal allograft recipients. *Transplantation* 1989; **48**: 800–805.
32. Perkins JD, Nelson DL, Rakela J, Grambsch PM, Krom RA. Soluble interleukin-2 receptor level as an indicator of liver allograft rejection. *Transplantation* 1989; **47**: 77–81.
33. Humbert M, Emilie D, Cerrina J, *et al.* Soluble interleukin 2 receptor and neopterin serum levels after lung/heart-lung transplantations — absence of predictive value for late allograft rejection. *Transplantation* 1991; **52**: 1092–1094.
34. Gascoigne AD, Shenton BK, White MD, Colquhoun IW, Dark JH, Corris PA. The value of plasma-soluble interleukin 2 receptor monitoring in lung transplantation. *Transplantation* 1993; **56**: 1029–1031.
35. Young Fadok TM, Simpson MA, Madras PN, Dempsey RA, O'Connor K, Monaco AP. Predictive value of pretransplant IL-2 levels in kidney transplantation. *Transplant Proc* 1991; **23**: 1295–1296.
36. Cohen N, Gumbert M, Birnbaum J, *et al.* An improved method for the detection of soluble interleukin 2 receptors in liver transplant recipients by flow cytometry. *Transplantation* 1991; **51**: 417–421.
37. Tono T, Monden M, Yoshizaki K, *et al.* Biliary interleukin 6 levels as indicators of hepatic allograft rejection in rats. *Transplantation* 1992; **53**: 1195–1201.
38. Gajewski TF, Joyce J, Fitch FW. Antiproliferative effect of IFN-gamma in immune regulation. III. Differential selection of TH1 and TH2 murine helper T lymphocyte clones using recombinant IL-2 and recombinant IFN-gamma. *J Immunol* 1989; **143**: 15–22.
39. Le Gros G, Ben Sasson SZ, Seder R, Finkelman FD, Paul WE. Generation of interleukin 4 (IL-4)-producing cells *in vivo* and *in vitro*: IL-2 and IL-4 are required for *in vitro* generation of IL-4-producing cells. *J Exp Med* 1990; **172**: 921–929.
40. Swain SL, Weinberg AD, English M, Huston G. IL-4 directs the development of Th2-like helper effectors. *J Immunol* 1990; **145**: 3796–3806.
41. Betz M, Fox BS. Regulation and development of cytochrome c-specific IL-4-producing T cells. *J Immunol* 1990; **145**: 1046–1052.
42. Swain SL, Huston G, Tonkonogy S, Weinberg A. Transforming growth factor-beta and IL-4 cause helper T cell precursors to develop into distinct effector helper cells that differ in lymphokine secretion pattern and cell surface phenotype. *J Immunol* 1991; **147**: 2991–3000.
43. Dallman MJ, Montgomery RA, Larsen CP, Wanders A, Wells AF. Cytokine gene expression: analysis using Northern blotting, polymerase chain reaction and *in situ* hybridization. *Immunol Rev* 1991; **9**: 163–179.
44. Dallman MJ, Larsen CP, Morris PJ. Cytokine gene transcription in vascularised organ grafts: analysis using semiquantitative polymerase chain reaction. *J Exp Med* 1991; **174**: 493–496.
45. Morgan C, Pelletier R, Hernandez C, *et al.* Cytokine mRNA expression during development of acute rejection in murine cardiac allografts. *Transplant Proc* 1993; **25**: 114–116.
46. Vandenbroecke C, Caillat Zucman S, Legendre C, *et al.* Differential *in situ* expression of cytokines in renal allograft rejection. *Transplantation* 1991; **51**: 602–609.
47. Morel D, Normand E, Lemoine C, *et al.* Tumor necrosis factor alpha in human kidney transplant rejection — analysis by *in situ* hybridization. *Transplantation* 1993; **55**: 773–777.
48. Martinez OM, Krams SM, Sterneck M, *et al.* Intragraft cytokine profile during human liver allograft rejection. *Transplantation* 1992; **53**: 449–456.
49. Dallman MJ, Roake J, Hughes D, Toogood G, Morris PJ. Sequential analysis of IL-2 gene transcription in renal transplants. *Transplantation* 1992; **53**: 683–685.
50. Nast CC, Zuo XJ, Prehn J, Danovitch GM, Wilkinson A, Jordan SC. Gamma-interferon gene expression in human renal allograft fine-needle aspirates. *Transplantation* 1994; **57**: 498–502.
51. Wood KJ, Evins J, Morris PJ. Suppression of renal allograft rejection in the rat by class I antigens on purified erythrocytes. *Transplantation* 1985; **39**: 56–62.
52. Cobbold SP, Martin G, Waldmann H. The induction of skin graft tolerance in major histocompatibility complex-mismatched or primed recipients: primed T cells can be tolerized in the periphery with anti-CD4 and anti-CD8 antibodies. *Eur J Immunol* 1990; **20**: 2747–2755.
53. Pearson TC, Darby CR, Bushell AR, West LJ, Morris PJ, Wood KJ. The assessment of transplantation tolerance induced by anti-CD4 monoclonal antibody in the murine model. *Transplantation* 1993; **55**: 361–367.
54. Wood KJ, Hopley A, Dallman MJ, Morris PJ. Lack of correlation between the induction of donor class I and class II major histocompatibility complex antigens and graft rejection. *Transplantation* 1988; **45**: 759–767.
55. Rush DN, Henry SF, Jeffery JR, Schroeder TJ, Gough J. Histological findings in early routine biopsies of stable renal allograft recipients. *Transplantation* 1994; **57**: 208–211.
56. Dallman MJ, Shiho O, Page TH, Wood KJ, Morris PJ. Peripheral tolerance to alloantigen results from altered regulation of the interleukin 2 pathway. *J Exp Med* 1991; **173**: 79–87.
57. Bugeon L, Cuturi MC, Hallet MM, Paineau J, Chabannes D, Soulillou JP. Peripheral tolerance of an allograft in adult rats — characterization by low interleukin-2 and interferon-gamma mRNA levels and by strong accumulation of major histocompatibility complex transcripts in the graft. *Transplantation* 1992; **54**: 219–225.
58. Takeuchi T, Lowry RP, Konieczny B. Heart allografts in murine systems. The differential activation of Th2-like effector cells in peripheral tolerance. *Transplantation* 1992; **53**: 1281–1294.
59. Broski AP, Halloran PF. Tissue distribution of IL-10 mRNA in normal mice. Evidence that a component of IL-10 expression is T and B cell-independent and increased by irradiation. *Transplantation* 1994; **57**: 582–592.
60. Field EH, Rouse TM, Fleming AL, Jamali I, Cowdery JS. Altered IFN-gamma and IL-4 pattern lymphokine secretion in mice partially depleted of CD4 T cells by anti-CD4 monoclonal antibody. *J Immunol* 1992; **149**: 1131–1137.
61. Papp I, Wieder KJ, Sablinski T, *et al.* Evidence for functional heterogeneity of rat CD4+ T cells *in vivo*. Differential expression of IL-2 and IL-4 mRNA in recipients of cardiac allografts. *J Immunol* 1992; **148**: 1308–1314.
62. Siegling A, Lehmann M, Riedel H, *et al.* A nondepleting anti-rat CD4 monoclonal antibody that suppresses T helper 1-like but not T helper 2-like intragraft lymphokine secretion induces long-term survival of renal allografts. *Transplantation* 1994; **57**: 464–467.
63. Maeda H, Takata M, Takahashi S, Ogoshi S, Fujimoto S. Adoptive transfer of a t(h)2-like cell line prolongs MHC class-II antigen disparate skin allograft survival in the mouse. *Int Immunology* 1994; **6**: 855–862.
64. Bierer BE, Hollander G, Fruman D, Burakoff SJ. Cyclosporin A and FK506: molecular mechanisms of immunosuppression and probes for transplantation biology. *Curr Opin Immunol* 1993; **5**: 763–773.
65. Rosenberg AS, Finbloom DS, Maniero TG, Van der Meide PH, Singer A. Specific prolongation of MHC class II disparate skin allografts by *in vivo* administration of anti-IFN-gamma monoclonal antibody. *J Immunol* 1990; **144**: 4648–4650.
66. Sakagami K, Ohsaki T, Ohnishi T, Saito S, Matsuoka J, Orita K. The effect of anti-interleukin 2 monoclonal antibody treatment on the survival of rat cardiac allograft. *J Surg Res* 1989; **46**: 262–266.
67. Imagawa DK, Millis JM, Olthoff KM, *et al.* The role of tumor necrosis factor in allograft rejection. II. Evidence that antibody therapy against tumor necrosis factor-alpha and lymphotoxin enhances cardiac allograft survival in rats. *Transplantation* 1990; **50**: 189–193.
68. Imagawa DK, Millis JM, Seu P, *et al.* The role of tumor necrosis factor in allograft rejection. III. Evidence that anti-TNF antibody therapy prolongs allograft survival in rats with acute rejection. *Transplantation* 1991; **51**: 57–62.
69. Bolling SF, Kunkel SL, Lin H. Prolongation of cardiac allograft survival in rats by anti-TNF and cyclosporine combination therapy. *Transplantation* 1992; **53**: 283–286.
70. Didlake RH, Kim EK, Sheehan K, Schreiber RD, Kahan BD. Effect of combined anti-gamma interferon antibody and cyclosporine therapy on cardiac allograft survival in the rat. *Transplantation* 1988; **45**: 222–223.
71. Paineau J, Priestley C, Fabre J, *et al.* Effects of gamma interferon and interleukin-2, and of gamma-interferon antibodies, on the rat immune response against allografts. *Transplant Proc* 1989; **21**: 999–1001.
72. Gugenheim J, Tovey M, Gigou M, *et al.* Prolongation of heart allograft survival in rats by interferon-specific antibodies and low dose cyclosporin A. *Transplant Int* 1992; **5** (Suppl 1): S460–S461.
73. Schorle H, Holtschke T, Hunig T, Schimpl A, Horak I. Development and function of T cells in mice rendered interleukin-2 deficient by gene targeting. *Nature* 1991; **352**: 621–624.
74. Kuhn R, Rajewsky K, Muller W. Generation and analysis of interleukin-4 deficient mice. *Science* 1991; **254**: 707–710.
75. Waldmann TA. The structure, function, and expression of interleukin-2 receptors on normal and malignant lymphocytes. *Science* 1986; **232**: 727–732.
76. Kupiec Weglinski JW, Tilney NL, Stunkel KG, *et al.* Agonistic and antagonistic interactions of anti-interleukin 2 receptor monoclonal antibodies in rat recipients of cardiac allografts. *Transplantation* 1989; **47**: 11–16.
77. Stunkel KG, Grutzmann R, Diamantstein T, Kupiec Weglinski JW, Schlumberger HD. Anti-interleukin-2 receptor monoclonal antibody therapy in rats: comparison of the effector mechanisms mediated by variant murine isotypes. *Transplant Proc* 1989; **21**: 1003–1005.
78. Tellides G, Dallman MJ, Morris PJ. Mechanism of action of interleukin-2 receptor (IL-2R) monoclonal antibody (MAb) therapy: target cell depletion or inhibition of function? *Transplant Proc* 1989; **21**: 997–998.
79. Kirkman RL, Barrett LV, Gaulton GN, *et al.* The effect of anti-interleukin-2 receptor monoclonal antibody on allograft rejection. *Transplantation* 1985; **40**: 719–722.
80. Ferrara JL, Marion A, McIntyre JF, Murphy GF, Burakoff SJ. Amelioration of acute graft vs host disease due to minor histocompatibility antigens by *in vivo* administration of anti-interleukin 2 receptor antibody. *J Immunol* 1986; **137**: 1874–1877.
81. Diamantstein T, Mouzaki A, Osawa H, *et al.* Inhibition of allograft rejection and organ-specific autoimmune disease by anti-interleukin-2 receptor (IL-2R)-targeted immunotherapy. The action of anti-IL-2R monoclonal antibodies and synergistic effect of cyclosporin A. *Dev Biol Stand* 1988; **69**: 177–184.
82. Kirkman RL, Shapiro ME, Carpenter CB, *et al.* A randomized prospective trial of anti-Tac monoclonal antibody in human renal transplantation. *Transplantation* 1991; **51**: 107–113.
83. Soulillou JP, Cantarovich D, Le Mauff B, *et al.* Randomized controlled trial of a monoclonal antibody against the interleukin-2 receptor (33B3.1) as compared with rabbit antithymocyte globulin for prophylaxis against rejection of renal allografts [see comments]. *N Engl J Med* 1990; **322**: 1175–1182.
84. Hiesse C, Kriaa F, Alard P. *et al.* Prophylactic use of the IL-2 receptor-specific monoclonal antibody LO-Tact-1 with cyclosporin A and steroids in renal transplantation. *Transplant Int* 1992; **5** (Suppl 1): S444–S447.
85. Brown PSJ, Parenteau GL, Dirbas FM, *et al.* Anti-Tac-H, a humanized antibody to the interleukin 2 receptor, prolongs primate cardiac allograft survival. *Proc Natl Acad Sci USA* 1991; **88**: 2663–2667.
86. Kirkman RL, Bacha P, Barrett LV, Forte S, Murphy JR, Strom TB. Prolongation of cardiac allograft survival in murine recipients treated with a diphtheria toxin-related interleukin-2 fusion protein. *Transplantation* 1989; **47**: 327–330.
87. Lorberboum Galski H, Barrett LV, Kirkman RL, *et al.* Cardiac allograft survival in mice treated with IL-2-PE40. *Proc Natl Acad Sci USA* 1989; **86**: 1008–1012.
88. Fanslow WC, Sims JE, Sassenfeld H, *et al.* Regulation of alloreactivity *in vivo* by a soluble form of the interleukin-1 receptor. *Science* 1990; **248**: 739–742.
89. Fanslow WC, Clifford KN, Park LS, *et al.* Regulation of alloreactivity *in vivo* by IL-4 and the soluble IL-4 receptor. *J Immunol* 1991; **147**: 535–540.
90. Haak Frendscho M, Marsters SA, Mordenti J, *et al.* Inhibition of TNF by a TNF receptor immunoadhesion. Comparison to an anti-TNF monoclonal antibody. *J Immunol* 1994; **152**: 1347–1353.
91. Mohler KM, Torrance DS, Smith CA, *et al.* Soluble tumor necrosis factor (TNF) receptors are effective therapeutic agents in lethal endotoxemia and function simultaneously as both TNF carriers and TNF antagonists. *J Immunol* 1993; **151**: 1548–1561.
92. Fernandez Botran R. Soluble cytokine receptors: their role in immunoregulation. *FASEB J* 1991; **5**: 2567–2574.
93. Hsieh CS, Heimberger AB, Gold JS, O'Garra A, Murphy KM. Differential regulation of T helper phenotype development by interleukins 4 and 10 in an alpha beta T-cell-receptor transgenic system. *Proc Natl Acad Sci USA* 1992; **89**: 6065–6069.
94. Fiorentino DF, Zlotnik A, Mosmann TR, Howard M, O'Garra A. IL-10 inhibits cytokine production by activated macrophages. *J Immunol* 1991; **147**: 3815–3822.
95. Sadick MD, Heinzel FP, Holaday BJ, Pu RT, Dawkins RS, Locksley RM. Cure of murine leishmaniasis with anti-interleukin 4 monoclonal antibody. Evidence for a T cell-dependent, interferon gamma-independent mechanism. *J Exp Med* 1990; **171**: 115–127.
96. Chatelain R, Varkila K, Coffman RL. IL-4 induces a Th2 response in Leishmania major-infected mice. *J Immunol* 1992; **142**: 1182–1187.
97. Silva JS, Morrissey PJ, Grabstein KH, Mohler KM, Anderson D, Reed SG. Interleukin 10 and interferon gamma regulation of experimental *Trypanosoma cruze* infection. *J Exp Med* 1992; **175**: 169–174.
98. Goldman M, Velu T. Interleukin-10 as an antiinflammatory and immunosuppressive cytokine. Abstract presented at the International conference on new trends in clinical and experimental immunosuppression, Geneva, 1994.
99. Brok HP, Heidt PJ, van der Meide PH, Zurcher C, Vossen JM. Interferon-gamma prevents graft-versus-host disease after allogeneic bone marrow transplantation in mice. *J Immunol* 1993; **151**: 6451–6459.
100. Liu Y, Linsley PS. Costimulation of T-cell growth. *Curr Opin Immunol* 1992; **4**: 265–270.
101. Jenkins MK, Johnson JG. Molecules involved in T-cell costimulation. *Curr Opin Immunol* 1993; **5**: 361–367.
102. Schwartz RH. A cell culture model for T lymphocyte clonal anergy. *Science* 1990; **24**: 1349–1356.
103. Jenkins MK, Mueller D, Schwartz RH, *et al.* Induction and maintenance of anergy in mature T cells. *Adv Exp Med Biol* 1991; **292**: 167–176.
104. Jenkins MK, Pardoll DM, Mizuguchi J, Chused TM, Schwartz RH. Molecular events in the induction of a nonresponsive state in interleukin 2-producing helper T-lymphocyte clones. *Proc Natl Acad Sci USA* 1987; **84**: 5409–5413.
105. Williams ME, Shea CM, Lichtman AH, Abbas AK. Antigen receptor-mediated anergy in resting T lymphocytes and T cell clones. Correlation with lymphokine secretion patterns. *J Immunol* 1992; **149**: 1921–1926.
106. Gilbert KM, Weigle WO. B cell presentation of a tolerogenic signal to Th clones. *Cell Immunol* 1992; **139**: 58–71.
107. Lenschow DJ, Bluestone JA. T cell co-stimulation and *in vivo* tolerance. *Curr Opin Immunol* 1993; **5**: 747–752.
108. Turka LA, Linsley PS, Lin H, *et al.* T-cell activation by the CD28 ligand B7 is required for cardiac allograft rejection *in vivo*. *Proc Natl Acad Sci USA* 1992; **89**: 11102–11105.
109. Lenschow DJ, Zeng Y, Thistlethwaite JR, *et al.* Long-term survival of xenogeneic pancreatic islet grafts induced by CTLA4lg [see comments]. *Science* 1992; **257**: 789–792.

ACKNOWLEDGEMENTS. We thank Professor Dr R. Benner and G. J. M. Tibbe for critical review of the manuscript. A.V. is supported by the Dutch Kidney Foundation.

**Received 22 August 1994; accepted 5 September 1994**
